# Health-risk behaviours among university students in Bulawayo, Zimbabwe

**DOI:** 10.3389/fpubh.2026.1914098

**Published:** 2026-10-09

**Authors:** Grace Moyo, Fortunate T. Silinda

**Affiliations:** Department of Psychology, University of South Africa, Pretoria, South Africa

**Keywords:** health-risk behaviours, peer influence, qualitative descriptive case study, socio-ecological perspective, university students

## Abstract

**Background:**

University students frequently engage in health-risk behaviours that may affect academic engagement, wellbeing, and campus experiences. Quantitative studies dominate the literature, leaving limited qualitative insight into how students in specific African university settings understand these behaviours and the contexts in which they occur.

**Methods:**

A single-site descriptive qualitative case study was conducted at a university in Bulawayo, Zimbabwe. Fifteen students aged 19–25 years participated in semi-structured telephone interviews. Five institutional documents provided supplementary contextual information. Data were analysed thematically using QDA Miner Lite and interpreted through a socio-ecological lens.

**Results:**

Students described six interconnected health-risk behaviours: substance use, smoking and waterpipe use, risky sexual behaviours, physical inactivity, unhealthy dietary behaviours cancel practices, and inadequate and irregular sleep. These behaviours were understood as shaped by interacting peer, academic, financial, psychological, and institutional influences rather than as isolated individual choices. Some accounts reflected personal experiences, while others concerned behaviours observed among peers. Perceived consequences include difficulties with concentration and attendance, emotional wellbeing, physical wellbeing, and social relationships. Participants advocated accessible counselling, peer education, health information, healthier coping strategies, and student-informed institutional support.

**Conclusion:**

Health-risk behaviours among these students were embedded in interconnected personal, social, economic, academic, and institutional contexts. The findings highlight the importance of multi-level, student-informed approaches to university health promotion that address both individual coping and the broader conditions shaping health behaviours. Further multi-site qualitative and mixed-methods research is needed across diverse Zimbabwean and Southern African university settings.

## 1 Introduction

Health-risk behaviours among university students such as, substance use, smoking and waterpipe use,risky sexual behaviours, physical inactivity,unhealthy dietary behaviours, and inadequate or irregular sleep, remain a significant public health and educational concern. These behaviours affect physical and psychological wellbeing and shape academic engagement and campus experience. The transition to university intensifies independence, peer influence, academic pressure, and exposure to new social environments, creating conditions in which such behaviours can emerge or intensify (1–3).

Existing research has largely relied on quantitative approaches and often examines health-risk behaviours individually (4). Far less is known about how students themselves interpret these behaviours within the everyday realities of specific African university settings. Zimbabwe provides an important context for examining these issues, given documented concerns about substance use and the socioeconomic challenges affecting the country (1). This qualitative descriptive case study addresses this gap. Guided by an interpretivist paradigm and a socio-ecological perspective, it explores students’ descriptions of health-risk behaviours, the factors they perceive as shaping these behaviours, the implications they associate with university life, and the strategies they consider appropriate at a selected university in Bulawayo.

### 1.1 Aim of the study

The aim of this study was to explore health-risk behaviours among university students at a selected university in Bulawayo, Zimbabwe, with particular attention to students’ experiences, perceptions, and views regarding the factors associated with these behaviours and their perceived implications for university life.

### 1.2 Scope statement

This study focuses on selected health-risk behaviours among university students in Zimbabwe, namely substance use, smoking and waterpipe use, physical inactivity, unhealthy dietary behaviours, inadequate and irregular sleep, and risky sexual behaviours. Guided by an interpretivist paradigm and a socio-ecological perspective, the study examines how students understand and describe these behaviours within the university context and the factors they perceive as shaping their engagement in them. The study draws on student accounts and relevant institutional documents to provide contextual insight into how these behaviours are experienced and interpreted. The scope is limited to the participating university and students who met the study’s eligibility criteria; therefore, the findings are intended to provide context-specific insights into health-risk behaviours within the university setting.

### 1.3 Research objectives

The study seeks to:

1. Identify the health-risk behaviours described by university students at a selected university in Bulawayo, Zimbabwe.
2. Examine students’ perceptions of the factors influencing health-risk behaviours within the university context.
3. Examine students’ perceptions of the effects of health-risk behaviours on their university experiences and views on strategies for addressing these behaviours.

### 1.4 Research questions

1. What health-risk behaviours are described by university students at a selected uinversity in Bulawayo, Zimbabwe?
2. What factors do students perceive as influencing these behaviours?
3. How do students perceive the effects of these behaviours on university experiences, and what strategies do they suggest?

## 2 Theoretical framing and literature review

### 2.1 Theoretical framing

The study is informed by a socio-ecological model adapted from McLeroy et al. (5). This perspective locates behaviour within nested layers: intrapersonal (knowledge, stress, coping), interpersonal (peers, social norms), institutional (policies, facilities, services), and broader structural conditions (financial precarity). The framework guided analysis and interpretation, allowing the findings to move beyond individual-deficit explanations.

### 2.2 Health-risk behaviours among university students

A substantial body of research has documented that university students engage in multiple health-risk behaviours, including substance use, smoking and waterpipe use, risky sexual practices, physical inactivity, unhealthy dietary patterns, and inadequate or irregular sleep (6–9). While quantitative studies have established the prevalence and correlates of these behaviours globally and in sub-Saharan Africa, qualitative insight into how students themselves describe and interpret them remains limited, particularly within specific Zimbabwean university contexts (1, 10, 11).

#### 2.2.1 Substance use

Substance use, especially alcohol and other drugs, is one of the most consistently reported health-risk behaviours among university students. The transition to university, marked by increased independence, new peer networks, academic pressure, and changing routines, creates conditions that can facilitate experimentation and regular use (7, 12). Zimbabwean studies confirm that peer influence, social environments, stress, and the desire for belonging are central drivers (11–13). Qualitative work further shows that substance use is embedded in both personal circumstances and institutional contexts rather than being a purely individual choice (10). Although associations with academic difficulties such as poor concentration and absenteeism have been reported (6, 11), these links are best understood as part of a wider web of stressors rather than simple causation. Overall, literature establishes substance use as a significant behaviour, yet it offers limited insight into how students themselves narrate its place in their university lives.

#### 2.2.2 Smoking and waterpipe use

Smoking and waterpipe (shisha/hookah) use remain common among university students. International research consistently identifies peer influence, socialising, stress, and misperceptions of harm as key associated factors (14–16, 49). Waterpipe smoking is particularly social and is often perceived as less harmful than cigarettes, despite evidence to the contrary [Arshad et al., 2019; (17, 18)]. Smoking frequently co-occurs with alcohol and other substance use, suggesting shared social contexts rather than isolated habits (19). In Zimbabwe, smoking has been identified as one of several negative lifestyle practices shaped by both personal and institutional factors (10). While the prevalence and social nature of smoking are well documented, fewer studies explore how students interpret the behaviour within the rules and realities of their specific campus environment.

#### 2.2.3 Risky sexual behaviours

Risky sexual behaviours, including inconsistent condom use, multiple partnerships, and casual sex, are prevalent among university students in sub-Saharan Africa (20, 21). Peer norms, social expectations, substance use, and variable sexual-health knowledge shape these practices (1, 22, 23). Zimbabwean research shows that students often recognise risk in partners’ behaviour and concurrent partnerships, yet personal risk perception remains low (24). Importantly, knowledge alone rarely determines behaviour; decisions are negotiated within relationships and broader social environments (9, 23). Although quantitative studies have mapped the extent of risky sexual practices, qualitative accounts of how students describe and make sense of these behaviours in everyday campus life are still relatively scarce.

#### 2.2.4 Physical inactivity

Physical inactivity is widespread among university students and is shaped by more than individual motivation. Academic workloads, limited facilities, financial constraints, and competing demands reduce opportunities for exercise (25, 26). A recent Zimbabwean study found that poor exercise infrastructure and the physical effort required were the most commonly cited barriers, with food insecurity and mental-health difficulties further increasing the likelihood of perceiving exercise as difficult (26). Other local research notes that while universities may promote healthy lifestyles in principle, actual facilities often fall short (25). These findings demonstrate that physical inactivity is produced by the interaction of academic pressure, economic realities, and institutional provision, yet student perspectives on these barriers within particular campuses remain under-explored.

#### 2.2.5 Unhealthy dietary behaviours

Unhealthy dietary patterns among university students typically involve frequent consumption of fast food and processed snacks, irregular meals, high intake of sugary drinks, and low consumption of fruits and vegetables (27–29). Financial constraints, time pressure, peer influences, and the campus food environment strongly shape these practices (10, 30). In Zimbabwe, cafeteria offerings and food prices have been highlighted as important institutional influences (25, 31). While literature clearly links economic and environmental factors to poor dietary choices, it provides less insight into how students themselves experience and explain these constraints in their daily university lives.

#### 2.2.6 Inadequate and irregular sleep

Inadequate and irregular sleep is highly prevalent among university students and is driven primarily by academic demands, social activities, technology use, and stress (32, 33). A meta-analysis of African university students reported that approximately 63% experience poor sleep quality, with stress, academic year, and bedtime device use as key associated factors (32). Zimbabwean qualitative studies have identified inadequate sleep as a negative lifestyle practice among university students, with students linking sleep difficulties to academic demands, financial pressure, and stress (10, 34). However, less is known about how students themselves interpret the relationship between sleep disruption and their broader university experiences.

### 2.3 Influences on health-risk behaviours: a socio-ecological perspective

Existing research points to interacting influences that operate across multiple levels, consistent with a socio-ecological understanding of behaviour (5). At the interpersonal level, peer relationships are among the most consistent identified drivers. Peers can normalise substance use, smoking, and certain sexual practices, and the desire for belonging often outweighs individual caution (11, 12, 22). Social settings, including parties and informal gatherings, further reinforce these norms. At the intrapersonal and academic level, stress, academic workload, and coping strategies feature prominently. Heavy academic demands disrupt sleep, reduce opportunities for physical activity, and increase the appeal of substances as short-term relief (10, 32, 33). Knowledge of health risks alone is frequently insufficient to change behaviour when stress and competing priorities are high. At the structural and institutional level, financial constraints can shape dietary choices and limit participation in recreational activities (29). University environments can also both support and constrain healthier lifestyles. Although sports facilities and exercise activities are available, socioeconomic constraints and competing academic demands may influence students’ ability to participate consistently in health-promoting activities (10, 26). Collectively, these findings indicate that health-risk behaviours cannot be adequately understood through individual-level explanations alone.

### 2.4 Implications and strategies

Health-risk behaviours may have implications for students’ physical health, psychological well-being, social relationships, and experiences of university life. Previous studies have reported associations between unhealthy lifestyles practices and outcomes such as reduced well-being, psychological distress, difficulties with concentration, and academic challenges (6, 10, 32). However, students may interpret these effects differently depending on their circumstances. For example, inadequate sleep may be understood in relation to academic demands by some students, while others may associate it with social activities, technology use, or stress. Similarly, substance use may be understood in relation to peer influence, social belonging, or coping with academic and personal pressures (10, 11, 22). Students’ perceptions are therefore important for understanding the broader meaning of health-risk behaviours within university life. Qualitative research provides an opportunity for students to describe these experiences in their own terms and explain how they understand the relationship between health-risk behaviours and their academic, social, and personal experiences.

Strategies for addressing health-risk behaviours may operate at individual, peer, and institutional levels. Previous literature has identified health education, counselling, student-support services, physical-activity opportunities, healthier food environments, and sexual-health services as potential components of university health promotion (4, 6, 26). Zimbabwean research also supports an institutional approach to promoting healthier student lifestyles. Gwizo (25) identified academic programmes, cafeteria services, physical activities, social activities, and university support systems as areas that could contribute to healthier student experiences. Similarly, Shava et al. (26) highlighted exercise infrastructure and campus-wide health promotion as relevant responses to barriers to physical activity. However, less is known about how students themselves perceive the appropriateness, accessibility, and feasibility of strategies for addressing health-risk behaviours within their university context. Exploring these perceptions can provide context-specific insights into strategies that students consider relevant and practical.

Research from South Africa has also demonstrated the importance of students’ perceptions in informing university health services. Khumalo et al. (35) identified limited knowledge, negative perceptions, privacy concerns, and previous experiences as barriers to health-service utilisation and recommended awareness campaigns and youth-friendly services. Although previous research provides insight into potential approaches, the appropriateness of particular responses may vary across university settings. There is limited qualitative evidence on the strategies that university students themselves consider appropriate for addressing health-risk behaviours within their specific institutional contexts, particularly in Zimbabwe. Understanding students’ views can therefore provide context-specific information to inform student-support and health-promotion initiatives.

### 2.5 Research gap

Despite a robust quantitative evidence base documenting prevalence and correlates, three critical gaps remain. First, qualitative studies that prioritise students’ own meaning-making and lived experience of health-risk behaviours are still scarce, particularly in Zimbabwean university settings. Second, behaviours are typically investigated in isolation rather than as interconnected practices shaped by the same peer, academic, financial, and institutional conditions. Third, few studies have examined, within a single bounded university context, how students simultaneously describe the behaviours, the factors they perceive as influencing them, the effects they attribute to academic and social life, and the strategies they consider appropriate. This study addresses these gaps by providing a socio-ecologically informed qualitative case study of student perceptions at one university in Bulawayo, Zimbabwe.

## 3 Methodology

### 3.1 Research paradigm and design

The study was guided by an interpretivist paradigm, which emphasises understanding individuals’ subjective experiences, meanings, and perspectives within their social and cultural contexts (36, 37). A single-site descriptive case study design was employed to develop an in-depth understanding of health-risk behaviours within their real-life institutional context (38, 39). The selected university in Bulawayo, Zimbabwe, constituted the bounded case. The study did not aim to estimate statistical prevalence or establish causal relationships but rather to understand how students described and interpreted health-risk behaviours within the university context.

### 3.2 Participants and sampling

The study involved 15 university students aged 19–25 years who were enrolled at the selected university in Bulawayo, Zimbabwe. The age range falls within the period of emerging adulthood, which is characterised by identity exploration, increasing autonomy, and the development of patterns of health-related behaviour (40, 41). Participants were selected using homogeneous purposive sampling based on characteristics relevant to the study objectives (42, 50). The initial participants were identified by the researcher based on the predefined eligibility criteria. Snowball sampling was subsequently used to facilitate the recruitment of additional eligible participants through existing participant networks (38). Although snowball sampling may result in participants being recruited from similar social networks, it was considered appropriate for reaching students who were willing to discuss potentially sensitive health-related topics.

Participants were eligible if they were enrolled at the selected university, aged 19–25 years, able to communicate in English or Ndebele, and willing to provide informed consent. The sample size was guided by the principle of information power, which proposes that the more relevant and information-rich the sample is to the study aim, the fewer participants may be required (43). Data collection continued until the interviews provided sufficient depth and no substantively new codes or themes emerging from the later interviews.

The five institutional documents were selected using criterion sampling. Documents were considered suitable if they were official university materials, were available during the study period, and contained information relevant to the university context and the study objectives. The five documents were selected because they provided complementary contextual information on student expectations, academic support, institutional information, student life, social activities, and wellbeing. Together, the documents provided a broader understanding of the institutional environment in which students experienced and interpreted health-risk behaviours. The documents were used to provide contextual information and were not treated as participants or as additional cases.

### 3.3 Data collection

Data were collected through semi-structured telephone interviews and documentary analysis. An interview guide was developed from the study objectives and research questions and was piloted before the main data collection. Interviews were conducted in English or Ndebele according to participants’ preference and lasted approximately 30–60 min. Telephone interviews were used for practical and accessibility reasons; the absence of visual non-verbal cues is acknowledged as a limitation of this mode. With participants’ consent, interviews were audio-recorded and transcribed verbatim. Where interviews were conducted in Ndebele, they were translated into English by the researcher, with accuracy checked against the original recordings. The researcher reviewed the recordings against the transcripts to ensure accuracy, and member checking was undertaken to allow participants to clarify or verify their accounts (37, 44). The five institutional documents were reviewed to provide contextual information relevant to the research questions. Documentary evidence was used solely to describe the institutional context and was not treated as evidence of students’ actual engagement in particular health-risk behaviours (45).

### 3.4 Data analysis

Data were analysed using thematic analysis following Braun and Clarke’s (46) approach. The researcher became familiar with the interview transcripts and institutional documents, generated initial codes, grouped related codes, developed preliminary themes and sub-themes, and reviewed and refined the emerging themes against the original data (37, 46). QDA Miner Lite Version 5 was used to organise, code, retrieve, and manage the qualitative data. The software served as a data-management tool and did not replace the researcher’s interpretive role in developing and refining codes and themes (47).

The researcher was the sole coder; therefore, formal inter-coder reliability was not calculated. Credibility was supported through repeated engagement with the data, systematic coding, checking transcripts against the audio recordings, member checking, and ongoing reflexive engagement throughout the analytical process. The analysis also distinguished between participants direct personal experiences, their accounts of other students’ behaviours, and their general knowledge or opinions.

### 3.5 Researcher reflexivity and trustworthiness

The researcher recognised the potential influence of personal knowledge, assumptions, and experiences on data collection and interpretation. Reflexivity was therefore maintained throughout the research process through the use of a reflexive journal, which documented methodological decisions, assumptions, and reflections arising during data collection and analysis. The researcher also undertook refresher training in qualitative interviewing and research methods to strengthen methodological competence. Trustworthiness was considered in terms of credibility, transferability, dependability, and confirmability (48). Credibility was supported through probing during interviews, transcript verification, member checking, and sustained engagement with the data. Transferability was addressed through thick description of the study context, participants, sampling, and research procedures, enabling readers to assess the relevance of the findings to other contexts. Dependability was supported through systematic documentation of research and analytical procedures. Confirmability was enhanced through reflexive practice and by grounding interpretations in the interview and documentary data.

### 3.6 Ethical considerations

Ethical approval was obtained from the University of South Africa College of Human Sciences Ethics Committee (NHREC Registration No. Rec-240816-052). The study complied with applicable institutional and local ethical requirements. Written informed consent was obtained by email before the telephone interviews. Participation was voluntary, and participants could decline to answer questions or withdraw without penalty. Confidentiality was maintained through the use of participant codes (P1–P15) and secure handling of recordings, transcripts, and other research materials.

## 4 Results

### 4.1 Description of participants and documentary sources

The study involved 15 university students from a selected university in Bulawayo, Zimbabwe. Each student interview was assigned a case code (P1–P15). Five institutional documents were also analysed to provide supplementary contextual information. These documents were coded D1–D5 and comprised the Student Handbook (D1), Tutorials on E-resources (D2), Frequently Asked Questions (D3), Silo Tips Bulletin (D4), and Students Social Life (D5). Findings are organised around the research questions and interpreted through a socio-ecological lens. The documents were analysed to situate students’ accounts within the institutional environment. They were not treated as participants or as evidence of the prevalence of health-risk behaviours among students. The qualitative dataset therefore comprised 15 interview transcripts and five institutional documents analysed within a single case-study design (Table 1).

**Table 1:** Description of participants and documentary sources.

Source category	Code(s)	Number	Type	Content
Students	P1-P15	15	Interviews	Students accounts and perceptions of health-risk behaviours, influencing factors, perceived effects, and suggested responses.
Student handbook	D1	1	Document analysis	Institutional information, student expectations, rules, services and campus activities.
Tutorials on E-resources	D2	1	Document analysis	Information on electronic learning resources and academic support.
Frequently asked questions	D3	1	Document analysis	Frequently asked student questions and institutional information provided in response.
Silo tips bulletin	D4	1	Document analysis	Student-oriented information and guidance relevant to student life and wellbeing.
Students social life	D5	1	Document analysis	Information concerning student social activities and aspects of student life.

Source: Field data (2023).

### 4.2 Participant characteristics

The participants were aged between 19 and 25 years. Nine participants were male and six were female. Four participants were in their first year, three were in their second year, two were in their third year, and six were in their fourth year. Thirteen identified as African and two as Coloured. These characteristics describe the participants included in the study and are not intended to imply statistical representation or generalisability.

The demographic characteristics presented in Table 2 were considered in interpreting the participants’ accounts. The health-risk behaviours identified in the interviews were reported across participants of different genders, ages, years of study and race. The participants’ accounts did not suggest that particular health-risk behaviours were exclusively experienced or described by a specific demographic group. For this reason, the findings were presented thematically rather than by demographic subgroup, while demographic characteristics were considered where they provided relevant context for understanding participants’ experiences and perceptions.

**Table 2:** Demographic characteristics of participants (*N* = 15).

Characteristics category	Number	Percentage %
Gender		
Male	9	60%
Female	6	40%
Age		
19–20	3	20%
21–23	5	33%
24–25	7	47%
Year of study		
First year	4	27%
Second year	3	20%
Third year	2	13%
Fourth year	6	40%
Race		
African	13	87%
Coloured	2	13%

Source: Field data (2023) Percentages are rounded.

### 4.3 Health-risk behaviours identified in the student accounts

The analysis identified six principal categories of health-risk behaviour: substance use, smoking, physical inactivity, unhealthy dietary behaviours, inadequate and irregular sleep, and risky sexual behaviours. Three additional issues, mental-health concerns, stress and poor stress management, and hygiene and self-care, also emerged across the accounts. Participants did not describe these behaviours uniformly. Some reported personal experiences, whereas others discussed behaviours observed among peers. This distinction is important because the findings capture both participants’ lived experiences and their perceptions of the wider student environment.

The behaviours also appeared interconnected. Substance use and smoking were discussed in relation to peer influence and stress management. Dietary practices were associated with financial circumstances and, on some accounts, body-image concerns. Sleep difficulties were linked to academic functioning, while risky sexual behaviours were discussed in relation to peer norms and academic engagement (Table 3).

**Table 3:** Health-risk behaviour themes identified in the qualitative dataset.

Health-risk behaviour/theme	Nature of evidence
Substance use	Personal accounts and observations of peers
Smoking	Personal accounts and observations of peers
Unhealthy dietary behaviours	Participant accounts
Physical Inactivity	Participant accounts
Inadequate and irregular sleep	Participant accounts
Risky sexual behaviours	Predominantly observations/perceptions of peer behaviour
Mental health concerns	Participant accounts and perceptions
Stress and coping	Participant accounts and perceptions
Hygiene and self-care	Participant accounts (minor theme)

Source: Field data (2023). The table presents themes identified through qualitative coding and does not represent behavioural prevalence. Institutional documents were treated as contextual sources only.

#### 4.3.1 Substance use

Substance use, particularly alcohol consumption, was prominent in the participants’ accounts. The accounts included both personal experiences and observations of substance use among other students. P8 described alcohol in terms of its immediate emotional effects: “Alcohol makes me feel good and high while allowing me to be in my world. I operate better when I’m intoxicated.” (P8) This account suggests that alcohol was perceived by the participant as providing temporary emotional relief and a sense of improved functioning. Alcohol was therefore not described solely as a health risk but also as having a perceived short-term benefit.

Peer influence was evident in P9’s account: “I consider myself a good boy though I allowed my friends to pressure me into their life of partying and getting drunk every weekend.” P9’s account indicates that social relationships influenced participation in drinking. The participant’s experience suggests that the desire to participate in peer activities could contribute to engagement in alcohol-related behaviour.

Other participants distinguished their own behaviour from substance use among peers. P15 stated that they consumed alcohol but did not smoke, while P1 reported no personal substance use but expressed concern about substance use among other students. P10 associated personal alcohol consumption with absenteeism, incomplete coursework, and delayed graduation. Taken together, the accounts indicate that substance use was understood in different ways across participants rather than as a uniform student behaviour. For some participants, alcohol was associated with temporary emotional relief, social interaction, or a perceived improvement in functioning. For others, substance use was associated with negative consequences for academic participation and progress. Peer relationships also shaped some participants’ accounts, particularly where drinking was connected to social participation and pressure to belong. These differences suggest that participants attached different meanings to substance use depending on their experiences and social contexts. Substance use was therefore described not only as health-risk behaviour, but also as social practice and, on some accounts, as a way of responding to emotional or social pressures.

The documentary evidence indicated that the university promoted a drug, alcohol, and tobacco-free environment. The findings therefore suggest a difference between formal institutional expectations and the behaviours discussed in some student accounts.

#### 4.3.2 Smoking

Smoking, including cigarettes, marijuana, and waterpipe smoking, was discussed as both personal experience and behaviour observed among peers. P4 described smoking as a longstanding behaviour: “I started smoking at a tender age while I was doing my secondary education … now it’s a challenge for me to do without.” (P4). This account indicates that smoking was not necessarily initiated within the university. Rather, it could continue from earlier stages of life into university.

In contrast, P10 rejected smoking because of its perceived health consequences. This difference suggests that awareness of health risks did not result in uniform behavioural choices among participants. P9 connected smoking and substance use with stress and the need for alternative coping strategies. This suggests that some participants understood smoking partly in relation to how students managed stress.

Overall, smoking emerged as both a social behaviour and a perceived coping response. The documentary evidence indicated that smoking and other substance use were prohibited within the university environment. Thus, as with substance use, students’ accounts reflected a tension between institutional expectations and behaviours discussed within the student environment.

#### 4.3.3 Physical inactivity

Physical inactivity was evident in several participants’ accounts. P1 stated: “I don’t exercise and now am failing even to use our lecture room stairs.” (P1) P4 similarly reported: “I rarely exercise. I find it hard.” P6 reported exercising occasionally and recognised the cognitive benefits of physical activity. These accounts show variation in participation. While some participants reported little or no exercise, others recognised its potential benefits.

The documentary evidence indicated that the university provided facilities for volleyball, basketball, soccer, and netball, as well as space for jogging and walking. The Student Handbook also indicated that a comprehensive sports day was held once each semester. The contrast between the availability of recreational facilities and participants’ reports of limited exercise is significant. It suggests that the availability of facilities alone did not guarantee participation.

#### 4.3.4 Unhealthy dietary behaviours

Participants described several dietary concerns, including junk-food consumption, energy-drink use, irregular meals, and limited access to balanced meals. P15 reported: “I take energy drinks excessively without having a proper meal. This has caused indigestion problems and inflammation in my stomach.” Financial circumstances were particularly prominent. P4 stated: “I eat a lot of junk food twice a day due to a lack of resources to buy proper food.” (P4) P6 similarly reported: “If you do not have money to buy food, you will eat only one meal in the evening.” (P6).

P10 also associated limited financial resources with difficulties maintaining regular meals and with reduced concentration. Body-image concerns were also evident. P5 described limiting food intake in an effort to maintain a slim body. This indicates that dietary behaviour was influenced not only by financial circumstances but also by perceptions of body image.

These findings suggest that unhealthy dietary practices cannot be understood solely as individual food choices. Participants’ accounts indicate that economic circumstances, food access, and body-image concern all formed part of the context in which dietary decisions were made.

The documentary evidence showed that food was available through the university cafeteria and that students were required to make individual arrangements and payments for meals. This provided contextual support for participants’ accounts concerning food access.

#### 4.3.5 Inadequate and irregular sleep

Inadequate and irregular sleep was associated with fatigue, concentration difficulties, academic challenges, stress, and perceived health changes.

P2 reported difficulty sleeping because of an irregular schedule and described daytime fatigue and difficulty concentrating. P3 similarly associated sleep deprivation with difficulty focusing, retaining information, and keeping up with academic work. P7 described perceived health effects associated with inadequate sleep, while P11 reported increased stress and mood changes. The excerpts indicate that participants viewed sleep as an important component of academic and psychological wellbeing. Sleep difficulties were described as affecting concentration, mood, and participation in academic activities. These findings represent participants’ reported experiences and perceptions. They do not establish that inadequate sleep directly caused the reported academic or health outcomes.

#### 4.3.6 Risky sexual behaviours

Risky sexual behaviours were discussed predominantly as behaviours observed among peers. Participants referred to unprotected sex, multiple sexual partners, and casual sexual relationships. P1 stated: “I’ve seen some of my peers engaging in risky sexual activities without thinking of the possible outcomes.” P5 referred to unprotected sex and casual relationships and perceived limited discussion of sexual health. P6 similarly described risky sexual behaviour as being normalised within some social groups and emphasised the importance of safe-sex education. These accounts suggest that participants perceived peer norms and limited sexual-health discussion as relevant to risky sexual behaviour. Participants also perceived possible academic implications. P11 associated preoccupation with sexual activities with reduced concentration and motivation, while P14 described friends whose sexual relationships appeared to compete with academic responsibilities.

Overall, the accounts suggest that risky sexual behaviour was understood primarily as part of the wider student social environment rather than as behaviour personally reported by most participants. Participants described perceived peer practices such as unprotected sex, casual relationships, and multiple partnerships, while also identifying limited discussion of sexual health as a concern. The emphasis on observed peer behaviour is important because it shows that participants interpreted sexual-health risks partly through what they saw and heard within their social networks. Some participants also associated these behaviours with reduced concentration or competing demands on students’ academic responsibilities. Thus, the findings provide insight into how students perceived and interpreted sexual-health practices within their social environment, rather than a direct account of participants’ own sexual behaviour.

#### 4.3.7 Cross-cutting issues

Three additional issues, mental-health concerns, stress and coping, and hygiene and self-care, cut across the main health-risk behaviour categories.

##### 4.3.7.1 Mental-health concerns

Mental health concerns emerged in several participants’ accounts. Participants referred to emotional difficulties, stress, mood changes, and psychological strain in relation to substance use, sleep difficulties, and academic pressures. The accounts suggest that mental health concerns were not experienced in isolation but were discussed alongside other aspects of student life. For example, alcohol was described by P8 as providing temporary emotional benefits, while P11 linked inadequate sleep with stress and mood changes. These findings suggest an interaction between health behaviours and psychological wellbeing in participants’ experiences. However, the study did not involve clinical assessment or diagnosis. The findings therefore represent participants’ reported experiences rather than evidence of clinically diagnosed mental-health conditions.

##### 4.3.7.2 Stress and coping

Stress and coping emerged across several accounts. Participants associated stress with academic and social pressures and, in some cases, with substance use and smoking. P9’s call for better coping strategies suggests that some participants perceived health-risk behaviours as ways of responding to stress. At the same time, participants recognised the need for healthier alternatives. Stress therefore appeared as a contextual issue that connected several of the health-risk behaviours rather than as isolated behaviour.

##### 4.3.7.3 Hygiene and self-care

Hygiene and self-care were identified less frequently than the other themes. Although the issue was not prominent, its presence indicates that participants understood health in broader terms that included everyday personal care. Because the theme occurred in only a small number of cases, it is treated as a supplementary finding rather than a major category of health-risk behaviour.

### 4.4 Factors perceived to influence health-risk behaviours

The findings identified four interconnected factors that participants associated with health-risk behaviours: peer influence and social context, stress and coping, financial circumstances, and institutional conditions.

#### 4.4.1 Peer influence and social context

Peer relationships were particularly evident in accounts of alcohol use and smoking. P9 described being pressured by friends to participate in partying and drinking. This suggests that social belonging could influence participation in certain health-risk behaviours. However, peer influence was not uniform. Some participants described observing risky behaviours among peers without reporting personal involvement. Peer influence therefore appeared to be an important contextual factor rather than a universal determinant.

#### 4.4.2 Stress and coping

Stress was repeatedly associated with substance use and smoking. P9’s call for alternative coping strategies suggests that participants perceived some health-risk behaviours as responses to pressure. The findings therefore indicate that coping was an important context in which some behaviours were understood. However, the qualitative accounts do not establish a causal relationship between stress and health-risk behaviours.

#### 4.4.3 Financial circumstances

Financial circumstances were particularly prominent in accounts of dietary behaviour. P4, P6 and P10 described financial difficulties affecting the availability and regularity of food. These findings suggest that economic circumstances could limit the choices available to students. Dietary behaviour was therefore influenced by structural conditions as well as individual preference.

#### 4.4.4 Institutional conditions

The university provided policies, recreational facilities, and food services intended to support student wellbeing. At the same time, participants’ accounts indicated that institutional resources did not always translate into student participation or compliance. For example, recreational facilities were available, yet some students reported limited physical activity. Similarly, the university had policies prohibiting substance use and smoking, while these behaviours continued to feature in student accounts. The findings therefore suggest that institutional policies and resources form an important context for student health behaviours but do not, by themselves, determine students’ practices.

### 4.5 Perceived effects on university experiences

Participants associated health-risk behaviours with academic participation, concentration, physical wellbeing, and psychological wellbeing. Alcohol use was associated with absenteeism and delayed academic progress in P10’s account. P9 also associated drinking with missing lectures. Sleep difficulties were linked to concentration and academic difficulties. P2 and P3 described problems with concentration and academic progress, while P7 and P11 described perceived physical and psychological effects. Dietary practices were also linked to physical and academic experiences. P15 reported physical discomfort associated with energy-drink consumption without adequate food, while P10 associated inadequate food intake with difficulty concentrating. These findings suggest that participants perceived health-risk behaviours as having effects that extended beyond physical health to academic and psychological domains. However, the findings represent participants’ perceptions and experiences. They should not be interpreted as demonstrating direct causal relationships between particular behaviours and particular outcomes.

### 4.6 Participants’ views on addressing health-risk behaviours

Participants suggested several approaches to addressing health-risk behaviours. These included improved coping strategies, sexual-health education, and greater participation in physical activities. P9 emphasised the importance of developing healthier coping strategies as alternatives to substance use and smoking. P6 highlighted the need for safe-sex education and increased awareness of the risks associated with unsafe sexual behaviour. The findings on physical inactivity also suggest that greater use of existing recreational facilities may be beneficial. The documentary evidence demonstrated that the university already had several recreational opportunities, although participants reported limited engagement. Overall, participants’ suggestions extended beyond individual behaviour change. Their accounts pointed to the importance of coping skills, peer relationships, health education, financial circumstances, and the effective use of institutional resources.

## 5 Discussion of findings

This study provides a context-specific qualitative account of how university students at one institution in Bulawayo understood health-risk behaviours. Students described multiple interconnected behaviours, including substance use, smoking, physical inactivity, unhealthy dietary practices, inadequate and irregular sleep, and risky sexual behaviours, alongside mental-health concerns, stress, and self-care. These behaviours were not described as isolated individual choices. Instead, participants situated them within peer relationships, academic pressures, financial constraints, and institutional conditions. Health-risk behaviours among university students have been shown to be influenced by both individual and social factors (10–12, 26). The present findings add contextual depth to this literature by showing how students interpreted these behaviours within their everyday university experiences. From an interpretivist standpoint, these accounts are important because they demonstrate that students attached different meanings to health-risk behaviours depending on their circumstances and social interactions.

### 5.1 Health-risk behaviours among university students

Substance use and smoking revealed a tension between perceived short-term benefits and longer-term consequences. Some participants described alcohol as providing temporary emotional or social relief, while others associated substance use and smoking with peer influence and social participation. Peer influence, social environments, and coping with stress have also been identified as important factors in substance use among university students (10, 11). The present findings help explain why these influences may be particularly important within university settings. Peer networks create opportunities for exposure and may shape perceptions of what is socially acceptable. However, exposure did not necessarily result in participation. Some participants described their own engagement, whereas others discussed behaviours they had observed among peers. This variation indicates that students did not respond to peer influence uniformly. Rather, they interpreted and negotiated these influences in relation to their own experiences. The findings therefore extend existing work by illustrating how peer influence and coping were understood within the specific social environment of the university.

Physical inactivity persisted despite the documented availability of recreational facilities. This suggests that institutional provision alone may not result in regular participation. Academic demands, motivation, and contextual barriers have been identified as factors that may limit physical activity among university students (26, 35). The present accounts help explain how these barriers may operate even when facilities are available. Participants appeared to negotiate opportunities for physical activity alongside academic responsibilities, established routines, and other demands. Thus, physical inactivity may reflect difficulties in incorporating activity into everyday university life rather than simply an absence of facilities. The finding highlights a gap between institutional provision and students’ actual engagement with available resources. It also shows that students interpreted physical activity in relation to competing priorities, rather than viewing participation solely as a matter of personal motivation.

Unhealthy dietary behaviours were strongly associated in participants’ accounts with financial constraints. Reliance on less nutritious foods, skipped meals, and limited access to balanced food suggest that dietary practices cannot always be explained by individual preference or knowledge. Participants described their food choices within financial circumstances that restricted the options they considered realistic. This finding adds contextual depth to the broader literature on student health behaviours by showing how material conditions may shape everyday dietary decisions. It also challenges explanations that place primary responsibility on individual knowledge or motivation. From an interpretivist perspective, the importance of this finding lies in understanding how students themselves made sense of their dietary choices. What may appear externally as an unhealthy choice could be understood by students as a practical response to their immediate circumstances.

Inadequate and irregular sleep was closely associated by participants with academic demands, fatigue, poor concentration, and psychological strain. Relationships between sleep and academic functioning among university students have been well established (6, 32). The present findings provide insight into how students experienced these relationships within everyday university life. Participants did not describe sleep only as an individual lifestyle practice. They connected sleeping patterns with academic schedules, stress, fatigue, and other responsibilities. This suggests that disrupted sleep was experienced within a wider process of managing competing academic and personal demands. The findings therefore contribute to existing research by showing how students understood sleep difficulties in relation to their broader university experiences.

Risky sexual behaviours were discussed predominantly as peer phenomena, including concerns about unprotected sex, multiple partners, casual relationships, limited sexual-health discussion, and the perceived normalisation of such behaviours within some social groups. Peer norms and social environments have been identified as important influences on health-related behaviours among young people and university students. The present findings provide further contextual insight by showing how students perceived and discussed sexual-health risks within their social networks. Importantly, many accounts referred to behaviours observed among peers rather than participants’ own experiences. These accounts therefore cannot be interpreted as evidence of the prevalence of risky sexual behaviours in the study population. Rather, they demonstrate how students constructed understandings of sexual-health risks through observation, conversations, and perceptions of peer norms. This distinction is consistent with the interpretivist orientation of the study, which focuses on participants’ meanings and interpretations rather than treating qualitative accounts as measures of behavioural prevalence.

Mental-health concerns emerged as a cross-cutting issue across several health-risk behaviour categories. Participants referred to emotional difficulties, stress, mood changes, and psychological strain in relation to substance use, sleep difficulties, and academic pressures. For example, alcohol was described by one participant as providing temporary emotional benefits, while inadequate sleep was associated with stress and changes in mood. These accounts suggest that participants did not experience physical and psychological wellbeing as separate aspects of university life. Instead, they described them as interconnected experiences shaped by academic and social pressures. Research has linked student health behaviours with psychological wellbeing and academic functioning (6, 32). The qualitative accounts add a student-centred dimension by showing how participants understood these connections. Because the study did not involve clinical assessment or diagnosis, these findings represent reported experiences and perceptions rather than clinically diagnosed mental-health conditions. This distinction also prevents the findings from being interpreted as evidence of causal relationships between health-risk behaviours and mental-health outcomes.

Hygiene and self-care emerged less frequently than the main health-risk behaviour categories but provided an additional perspective on how students understood health and wellbeing. Participants’ accounts suggest that their understanding of health extended beyond substance use, diet, physical activity, sleep, and sexual behaviour to include everyday practices of personal care. Although this theme was not sufficiently prominent to support extensive interpretation, it demonstrates that students’ understanding of wellbeing included routine practices of self-care. Given its limited prominence, however, hygiene and self-care should be regarded as a supplementary finding rather than a major pattern.

### 5.2 Interconnected influences on health-risk behaviours

The findings indicate that health-risk behaviours were experienced within several interconnected levels of students’ social environments. This interpretation corresponds with the socio-ecological framework, which recognises that health behaviours are influenced by interactions between individual, interpersonal, organisational, community, and broader structural factors (5). In the present study, peer relationships were particularly evident in accounts of substance use, smoking, and risky sexual behaviours. However, peer influence was not deterministic. Some participants described observing behaviours among peers without adopting them themselves. This suggests that social environments may shape exposure, opportunities, and perceptions of acceptable behaviour without producing uniform responses. The finding therefore adds an interpretive dimension to the socio-ecological framework by showing that students experienced and understood interpersonal influences differently.

Academic pressure and coping also featured prominently across participants’ accounts. Participants associated stress and academic demands with substance use, smoking, disrupted sleep, and reduced opportunities for physical activity. Academic and psychosocial pressures have been identified as relevant to student health behaviours (25, 26, 35). The present findings provide insight into how these pressures were experienced in everyday university life. Participants did not simply identify stress as an external influence. They described particular behaviours in relation to attempts to respond to or manage academic and emotional pressures. This suggests that the meaning of health-risk behaviour may partly depend on the circumstances in which students experience it. From an interpretivist perspective, coping is therefore important not only because of the behaviour itself but also because of how students understood its purpose within their circumstances.

Financial circumstances provided another important contextual influence, particularly in relation to dietary practices. Participants described financial limitations as restricting their ability to access balanced food. This illustrates how material conditions may constrain health-related choices rather than leaving students with unlimited alternatives. Institutional conditions also shaped students’ experiences. Although the university provided recreational facilities, food services, and policies addressing substance use and smoking, participants’ accounts indicated that institutional provision did not always result in behavioural uptake. Contextual influences on student health behaviours have been emphasised in the literature (25, 26, 35). The present findings extend this understanding by showing that the availability of a resource does not necessarily mean that students experience it as accessible, relevant, or manageable within their everyday circumstances.

Taken together, the findings challenge explanations that locate health-risk behaviours exclusively within individual decision-making. Students appeared to negotiate health-related behaviours within overlapping social, academic, economic, and institutional circumstances. The socio-ecological framework provides a basis for locating these influences across different levels (5), while the interpretivist paradigm directs attention to how students themselves understood and assigned meaning to those influences. The two perspectives therefore serve different but complementary purposes. The socio-ecological framework helps identify where influences operate, whereas interpretivism helps explain how students experienced and interpreted them. This combination provides a more context-sensitive understanding of health-risk behaviours than an explanation based solely on individual choice.

### 5.3 Perceived effects on academic and psychological wellbeing

Participants associated health-risk behaviours with academic participation, concentration, physical wellbeing, and emotional experiences. Alcohol use was described in relation to absenteeism and delayed academic progress, inadequate sleep with concentration difficulties, and inadequate dietary practices with physical discomfort and reduced focus. Student health behaviours, sleep, wellbeing, and academic functioning have been linked in the literature (6, 32). The present accounts add depth to these findings by showing how students experienced these relationships in their everyday lives. Rather than simply identifying behaviours as factors associated with academic functioning, participants described how particular behaviours fitted into their experiences of studying, coping, and maintaining wellbeing.

The qualitative accounts also show that these perceived effects were not understood uniformly. The same behaviour could carry different meanings for different students. For example, alcohol was described by one participant as providing temporary emotional relief, whereas another participant associated alcohol use with academic difficulties. This variation demonstrates that students did not interpret health-risk behaviours only in terms of their negative consequences. A behaviour could be perceived as providing an immediate social or emotional benefit while also being associated with longer-term difficulties. This finding is important because it demonstrates the complexity of students’ interpretations and shows why health-risk behaviours cannot be fully understood by considering their outcomes alone.

The findings further suggest that academic and psychological wellbeing were interconnected in participants’ accounts. Difficulties with sleep, substance use, stress, and dietary practices were discussed alongside concentration, mood, motivation, and academic participation. These accounts should not be interpreted as establishing direct causal relationships. Instead, they demonstrate how students perceive different aspects of their wellbeing as connected. This distinction is important because the study sought to understand participants’ experiences rather than clinically establish the effects of particular behaviours. The interpretation therefore remains consistent with the interpretivist paradigm by prioritising the meanings participants attached to their experiences.

### 5.4 Participants’ perspectives on addressing health-risk behaviours

Participants’ suggested responses extended beyond individual behaviour change. They emphasised healthier coping strategies as alternatives to substance use and smoking, accessible sexual-health education, and greater engagement with recreational opportunities. These suggestions reflect the contextual nature of the findings, in which health-risk behaviours were described within social, academic, and economic environments. Psychosocial and contextual influences have been highlighted as important in understanding student health behaviours (25, 35). The present findings add to this literature by showing that students themselves identified forms of support that they considered relevant to the circumstances in which these behaviours occurred.

The emphasis on coping strategies is particularly important. Where students interpreted substance use or smoking as responses to stress and pressure, information about health risks alone may not address the circumstances they identified as contributing to these behaviours. The findings therefore suggest that health promotion may be more meaningful when students are offered practical alternatives for managing stress and academic pressures. This interpretation moves beyond simply informing students about the consequences of risky behaviours and considers the circumstances in which those behaviours acquire meaning.

Similarly, the findings concerning diet and physical activity suggest that interventions should consider the conditions within which students make health-related decisions. Where financial constraints influence food choices, individual education may have limited effect if healthier options are not realistically accessible. Likewise, where recreational facilities are available but underused, universities may need to consider academic schedules, motivation, and students’ perceptions of accessibility rather than assuming that provision will automatically result in participation. These findings reinforce the importance of addressing contextual conditions alongside individual behaviour. Overall, the findings support multi-level health-promotion strategies that combine individual support with attention to peer norms, coping, economic circumstances, academic pressures, and institutional conditions. The socio-ecological framework provides a useful basis for understanding these multiple levels of influence (5). The interpretivist paradigm complements this framework by emphasising students’ own interpretations of their experiences. Accordingly, students should not be viewed simply as recipients of health information. Their perspectives indicate that health-related behaviours are interpreted and negotiated within the social and material realities of university life.

Overall, the discussion remains aligned with the interpretivist paradigm underpinning the study. The findings show that health-risk behaviours were understood through participants’ experiences, social interactions, and interpretations of university life rather than treated as fixed characteristics or purely individual choices. The same behaviour could carry different meanings depending on students’ circumstances, including coping, social participation, or perceived negative consequences. This emphasis on situated meaning strengthens the methodological coherence of the study. The socio-ecological framework identifies the multiple contexts in which health-risk behaviours occur, while the interpretivist paradigm explains how students made sense of those contexts. Together, these perspectives provide a context-sensitive interpretation of students’ accounts without overstating what the qualitative data can establish.

### 5.5 Contribution of the study

This study contributes a qualitative, context-specific examination of multiple health-risk behaviours, their perceived drivers, consequences, and preferred responses within a single Zimbabwean university setting. By foregrounding students’ own meaning-making and applying a socio-ecological lens, the study provides an integrated account of multiple health-risk behaviours and the social, economic, academic, and institutional contexts in which students described these behaviours. The findings offer grounded insights that can inform more responsive student health-promotion initiatives in similar higher-education environments.

### 5.6 Limitations of the study

First, the study was conducted at a university in Bulawayo. Institutional, social, and economic characteristics may differ elsewhere; findings should not be statistically generalised. Thick description of the context and participants is provided to support judgements of transferability. Second, the study relied on self-reported accounts of sensitive topics. Despite confidentiality measures, under-reporting or socially desirable responses remain possible.

Third, some participants described behaviours observed among peers rather than personal practices. Findings concerning certain behaviours, particularly risky sexual behaviour and some substance use, should therefore be read as perceptions of the wider student environment.

Fourth, the descriptive qualitative case study design prioritised depth and contextual understanding rather than prevalence estimation or causal inference. Findings are interpreted strictly as students’ descriptions and perceptions. Finally, institutional documents provided contextual information only; they do not independently establish actual student behaviour or the effectiveness of existing interventions.

## 6 Conclusion

This study explored how university students at one institution in Bulawayo, Zimbabwe, understood health-risk behaviours and the factors shaping them. Students described a range of interconnected behaviours, substance use, smoking, physical inactivity, unhealthy dietary practices, inadequate and irregular sleep, and risky sexual behaviours, alongside mental-health concerns, stress, and self-care. These behaviours were not understood as isolated individual choices but as practices shaped by interacting personal, social, economic, academic, and institutional conditions.

Peer relationships, academic demands, stress, financial constraints, and features of the university environment emerged as interconnected influences. Participants also described perceived effects on academic engagement, concentration, physical and emotional wellbeing, and social relationships. Importantly, some accounts reflected personal experiences, while others concerned behaviours observed among peers, providing insight into both lived experience and perceptions of the wider campus environment. Participants advocated multi-level responses that combine practical coping strategies, health education, peer support, and improved access to health-promoting resources. These findings underscore the importance of student-informed, socio-ecologically oriented approaches to campus health promotion. Rather than focusing solely on individual responsibility, health-promotion initiatives should also address the social, economic, academic, and institutional conditions that shape students’ health behaviours.

Overall, the study offers contextually grounded qualitative insights into health-risk behaviours within one Zimbabwean university. While the findings cannot be statistically generalised, they provide a useful foundation for strengthening student health-promotion initiatives and guiding further research in similar higher-education settings.

### 6.1 Recommendations

Based on the findings, the following recommendations are proposed:

1 Strengthen integrated student health and psychosocial support

The university should strengthen confidential health, counselling, and psychosocial support services that address substance use, stress, sleep difficulties, sexual health, and related wellbeing concerns. Clear referral pathways to specialised support should also be established.

2 Develop structured peer-education programmes

Structured peer-education programmes should be established or expanded to promote positive peer influence and healthier student norms. Peer educators can provide basic health information, encourage help-seeking, and refer students to professional services when needed.

3 Improve access to affordable nutritious food

Given participants’ accounts of financial constraints, skipped meals, and reliance on less nutritious options, the university should explore ways of improving students’ access to affordable and balanced food. Affordable meal options or targeted food-support initiatives should be considered where feasible.

4 Strengthen opportunities for physical activity

Although recreational facilities exist, participation remains limited. The university should increase student engagement through regular, inclusive recreational activities and programmes that accommodate diverse interests and schedules.

5 Involve students in health-promotion initiatives

Students should be systematically involved in the design, implementation, and evaluation of health-promotion programmes to ensure that interventions reflect their lived realities, priorities, and constraints.

6 Conduct further research

Further research should examine health-risk behaviours across multiple universities in Zimbabwe and the wider Southern African region. Qualitative, quantitative, or mixed-methods designs could further examine and refine the patterns identified in this single-site study.

## Data Availability

The original contributions presented in the study are included in the article/supplementary material, further inquiries can be directed to the corresponding author.
